# The threat of the spread of SARS-CoV-2 variants in animals

**DOI:** 10.1080/01652176.2021.2008046

**Published:** 2021-11-28

**Authors:** D. Katterine Bonilla-Aldana, Alfonso J. Rodriguez-Morales

**Affiliations:** a Semillero de Investigación en Zoonosis (SIZOO), Grupo de Investigación GISCA, Fundación Universitaria Autónoma de las Américas, Pereira, Risaralda, Colombia; b Universidad Científica del Sur, Lima, Perú; c Grupo de Investigación Biomedicina, Faculty of Medicine, Fundación Universitaria Autónoma de las Américas, Pereira, Risaralda, Colombia; d School of Medicine, Universidad Privada Franz Tamayo (UNIFRANZ), Cochabamba, Bolivia

Coronaviruses (CoVs) are enveloped, positive-sense, single-stranded RNA viruses. These viruses have adapted to infect many animal species, from bats to camels (Munir et al. 2020). Currently, seven CoVs may infect humans, of which three of them have caused epidemics, the Severe Acute Respiratory Syndrome type 1 (SARS-CoV-1), the Middle East Respiratory Syndrome (MERS-CoV) and the Severe Acute Respiratory Syndrome type 2 (SARS-CoV-2) (Rabaan et al. 2020), which is currently causing the Coronavirus Disease 2019 (COVID-19) pandemic.

The first case of SARS-CoV-1, which presented with atypical pneumonia, was documented at the end of 2002 in Guangdong, China. The SARS-CoV-1 epidemic caused 8,096 reported cases, with 774 deaths in many countries around the world. The MERS virus was first reported in Saudi Arabia in 2012, which caused only 2,521 cases with 866 deaths (Rabaan et al. 2020).

In December 2019, a series of human cases of severe pneumonia of unknown aetiology was detected in Wuhan, Hubei province, China (Zhu et al. 2020). The infection would be traced to the live animal wholesale market in that city. By March 11, 2020, the COVID-19 has declared a pandemic.

Concerning the disease, it has been considered a zoonotic origin. Among the studies that have been carried out, bats appear to be natural hosts of the virus (Cui et al. 2019). SARS-CoV-2 shares 96.2% identity at the nucleotide level with RaTG13, a CoV detected in species of horseshoe bats (*Rhinolophus sinicus*); this virus, however, has not been detected in humans (Zhou et al. 2020). Furthermore, the participation of other intermediate hosts, probably pangolins, as a conduit in the transmission of SARS-CoV-2 to humans cannot be excluded (Boni et al. 2020). In addition to its zoonotic origin, SARS-CoV-2 has been detected in animal hosts.

There are different experimental studies where they have demonstrated the susceptibility of SARS-CoV-2 in animals (Kim et al. 2020, Lu et al. 2020, Shi et al. 2020, van den Brand et al. 2008), in the same way, the detection of natural infections by the virus in animals has been presented, raising concern about reverse zoonosis also called zooanthroponosis (transmission of infection from humans to animals). In addition, several cases of felines, canines, zoo animals, minks and ferrets have tested positive for SARS-CoV-2, mainly due to close contact with infected humans (Bonilla-Aldana et al. 2021).

After mid-2020, significant mutations (such as the D614G) of the SARS-CoV-2 have been reported in humans, leading to the classification of the so-called variants of concern (VOC) and variants of interest (VOI). In the case of the VOCs, these are associated with an increase in transmissibility or detrimental change in covid-19 epidemiology; or increase in virulence or change in clinical disease presentation; or decrease in the efficacy and effectiveness of public health and social measures or available diagnostics, vaccines, therapeutics, according to the World Health Organization (WHO). A few weeks ago, in mid-2021, there were no reports and publications of VOCs and VOIs in animals. However, recently this has been reported and increased. For example, one of the studies mentioned the natural infection by SARS-CoV-2 in Asian lions (*Panthera leo persica*) caused by the lineage B.1.617.2 or Delta VOC, in which the lions showed signs of loss of appetite, serous nasal discharge and occasional cough (Karikalan et al. 2021; Mishra et al. 2021). Additionally, another study reported the first human-to-dog transmission caused by the Iota variant of SARS-CoV-2 in Latin America (Ricardo et al. 2021). In addition, recently, the pathogenicity of B.1.617.2 (Delta) and B.1.617.3 lineage of SARS-CoV-2 has been evaluated and compared with that of B.1, an early virus isolate with D614G mutation in a Syrian hamster model, showing that the Delta VOC may induce lung disease of moderate severity in about 40% of infected animals, which supports the attributed disease severity of the variant (Mohandas et al. 2021). Recently, a study of 26 canine and feline patients with suspected myocarditis at a veterinary referral centre in United Kingdom, found two cats and one dog positive by RT-PCR to SARS-CoV-2, sequencing the B.1.1.7 linage (Alpha VOC), also raising the question of its possible pathogenicity in these animals (Ferasin et al., 2021).

According to the above, strict biosecurity measures must be implemented for wild animals in captivity; in addition, close contact between humans infected with SARS-CoV-2 and pets must be prevented to avoid the appearance of new mutations of importance in public health due to zooanthroponosis events. The COVID-19 pandemic has not been over yet, and then, vaccination in humans and animals should be increased, as these are also susceptible in a considerable proportion (Bonilla-Aldana et al. 2021).
